# Drivers of China’s Industrial Carbon Emissions: Evidence from Joint PDA and LMDI Approaches

**DOI:** 10.3390/ijerph15122712

**Published:** 2018-12-01

**Authors:** Feng Dong, Xinqi Gao, Jingyun Li, Yuanqing Zhang, Yajie Liu

**Affiliations:** School of Management, China University of Mining and Technology, Xuzhou 221116, China; gaoxinqi1993@163.com (X.G.); 15852487068@163.com (J.L.); m18852254830@163.com (Y.Z.)

**Keywords:** carbon emissions, factor decomposition, LMDI, Shephard distance function, PDA, Chinese industry

## Abstract

As the world’s top carbon-emitting country, China has placed great emphasis on understanding the driving factors of carbon emissions and developing appropriate emissions reduction policies. Due to the obvious variations in carbon emissions among various industries in China, corresponding policies need to be formulated for different industries. Through data envelopment analysis, this study introduced the Shephard distance function into the logarithmic mean Divisia index (LMDI) for decomposition analysis, built a carbon emissions decomposition model of 23 industries in China during 2003–2015, and analyzed the impact of 10 factors driving carbon emissions. The main results are as follows. (1) Potential gross domestic production (GDP) is a crucial factor for increasing carbon emissions, whereas potential energy intensity and technological advances of carbon emissions have a significant inhibitory effect on carbon emissions; (2) the technological progress of energy usage and the technological advances of GDP output are manifested by inhibiting carbon emissions at the early stage of development and increasing emissions at the later stage; (3) the structure of coal-based energy consumption is difficult to change in the long term, resulting in a weak effect of energy mix on carbon emissions and an increase in carbon emissions due to the potential energy carbon intensity factor.

## 1. Introduction

Global warming has attracted increasing attention since the late 1980s. Environmental degradation caused by excessive fossil energy consumption has become increasingly salient [1], especially as the global economy has grown rapidly. Excessive emissions of greenhouse gases are responsible for warming the climate, and carbon dioxide accounts for the majority of greenhouse gases. Therefore, controlling the increase of carbon emissions has become a key measure to slow global warming. Since the 1990s, energy-related carbon dioxide emissions in newly industrialized countries have increased significantly compared with those in industrialized countries [2]. In 2007, China surpassed the United States of America to become the largest carbon-emitting country in the world [3,4].

Under pressure to reduce carbon emissions, China must shoulder its share of responsibility as one of the world’s leading economic and political powers and contribute to global energy conservation, carbon emissions reduction, and low-carbon development. China pledged to reduce carbon intensity by 60–65% compared with the level in 2005 [5,6,7], increase non-fossil energy to approximately 20% of primary energy consumption [8], and achieve peak carbon emissions in 2030 [9,10,11,12,13]. However, as industrialization and urbanization gather momentum and the economy develops vigorously, China will inevitably continue to consume a large amount of fossil-based energy, and thus will continue to generate a large amount of carbon emissions. Under such a grim situation, how to achieve win-win results for economic development and emissions reduction is one of the major tasks of the Chinese government.

Considering the severity of the problem, China’s traditional extensive and high-energy development mode should be changed in order to realize the low-carbon transformation of the industrial structure. The structure of the industrial sector plays an important role in the development of a low-carbon economy. The environmental pollution caused by economic development originates from the resources consumed and the emitted waste gas. The production process of different industries has different impacts on the ecological environment, and the CO_2_ emissions generated from production are also very different. In order to analyze the irrationality of the existing industrial structure and the emissions reduction path, this paper explored the specific differences of CO_2_ emissions in various industries in the process of economic development, and adopted the factor decomposition analysis method to understand the actual action mechanism of different factors on CO_2_ emissions in different industries, which is also the purpose of this paper to select 23 industries for research. Because some industries are a collection of similar industries, this paper divided the whole industry into 23 industries in the process of research.

When exploring the driving factors of carbon emissions through production-theoretical decomposition analysis (PDA), Chinese and other scholars have mostly concentrated on six economic sectors in China as a whole (or in regions) to formulate corresponding policies. This research extends previous studies by making the following contributions. (1) The PDA method was combined with the logarithmic mean Divisia index (LMDI) method to examine drivers of China’s industrial carbon emissions; (2) in-depth differences of carbon emissions and driving factors in many more different economic sectors were explored, and the number of research objects was increased to 23 segmented industries; (3) the PDA method was employed to create a 10-factor decomposition model of the carbon emissions resulting from energy consumption in 23 industries, which include potential energy carbon intensity, energy mix, and potential energy intensity; (4) by comparing the average changes of the whole industry with those of various industries, its action mechanism can be deeply explored.

This paper is organized as follows. In Section 2, we introduce the methodology and relative data. Results and discussion are presented in Section 3. Finally, we conclude this study.

## 2. Literature

At present, decomposition analysis is widely applied to examine various aspects of energy and the environment. Decomposition analysis quantitatively measures the contribution of each factor in a given process by decomposing the comprehensive or relative indicators into several driving factors. Three types of decomposition analysis are used to examine carbon emissions in China and internationally: index decomposition analysis (IDA), structural decomposition analysis (SDA), and production-theoretical decomposition analysis (PDA, a method that combines nonparametric distance functions and environmental production techniques) [14].

Due to its simple structure, easy-to-understand principles, and low data requirements [15], the IDA method has been widely applied by Chinese and international scholars to analyze the driving factors of energy consumption or carbon emissions from multiple dimensions and industries [12,16]. For example, Zhu et al. [17] employed the logarithmic mean Divisia index (LMDI, a type of IDA) and extended the Kaya [18] identity equation to examine the contribution of population size, economic output scale, industrial structure, energy mix, and energy efficiency to carbon emissions in China during 1980–2007. Li et al. [19] constructed a factor decomposition analysis model based on the period 1980–2007, and discovered that the factors causing increased carbon emissions are economic growth and industrial structure, while carbon emission intensity is an important factor in suppressing carbon emissions. Dong et al. [20] adopted the LMDI decomposition model to decompose the incremental changes in carbon emissions into four effects, two of which are economic scale and industrial structure. However, when researchers decompose carbon emissions via the IDA method, the emissions are typically decomposed into energy mix, energy intensity, population size, and economic output [21,22,23]. Although IDA has many advantages, such as low data requirements, variable form and easy understanding, it only considers the carbon emissions generated by direct energy consumption, and cannot explore the carbon emissions generated by indirect consumption. Meanwhile, this method cannot explore the amount or rate of contribution by changes of production technology to carbon emissions; hence, certain potential influencing factors are ignored.

Based on input‒output tables, SDA was employed to explore the factors that influence carbon emissions [24]. The SDA can explore the carbon emissions generated by direct and indirect energy sources. For example, Dong et al. employed the SDA to explore the influencing factors of carbon emission intensity in China from 1992 to 2012, and found that energy efficiency was an important driver of carbon emission intensity reduction [25]. Su et al. studied the drivers of Singapore’s carbon emissions using SDA, and found the expansions of export-oriented industries and export volume caused emissions to increase, and fuel switching and energy efficiency helped reduce emission growth [26]. Therefore, based on the SDA decomposition, the carbon emissions flow included in import and export can be examined [27,28]. However, because SDA-based research has a high data requirement, and moreover, input-output tables are not updated frequently, SDA is not conducive to in-depth research. Compared with IDA and SDA, PDA embeds the Shephard distance function into Kaya identity equation and introduces potential factors and technical efficiency into decomposition identities [29]. Through constructing the optimal production frontier, PDA comprehensively considers the influence of production efficiency and technology on energy use and carbon emissions [30].

Previous decomposition analyses have shown that energy intensity is a typical indicator of energy efficiency; however, it is difficult to propose targeted policy recommendations by merely replacing energy efficiency with energy intensity [31]. Because scholars have determined that production technology is a factor that affects carbon intensity and energy intensity [32,33,34,35], technical effects should be taken into consideration when studying the factors that drive carbon emissions. Some researchers have explored the effect of technology by combining decomposition methods with data envelopment analysis (DEA) methods [36,37,38]. Zhou and Ang [38] proposed PDA and applied them to analyze the carbon emissions in countries comprising the Organization for Economic Cooperation and Development.

Production-related factors play a very important role in regional carbon emissions. In production activities, economic entities produce both desired and undesired outputs. Technical efficiency influences the transformation of inputs into outputs, and thus directly influences production efficiency. Using the PDA framework, Kim and Kim [34] replaced the Malmquist index with the LMDI index and incorporated industrial structure and energy mix into the model, thus making up for the shortcomings in previous research by Zhou and Ang [38]. Zhang et al. [39] adopted PDA to decompose carbon emissions and used the generalized Fisher index for detailed decomposition. As a whole, PDA is a significant method for studying the impact of production technologies within various sectors, regions and countries on energy and carbon emissions, and more targeted policy recommendation can be proposed based on it [40]. The LMDI is widely applied in IDA decomposition because the index allows the zero value in decomposition [41] and data defect issues in decomposition are addressed very easily [42,43,44]. Therefore, in this study, a decomposition model that combined LMDI with PDA was employed.

Although existing research has played an important role in China’s energy conservation and emission reduction policies, there are still some deficiencies. First, most of the literature on carbon emissions focused on the national or regional level, which was incomplete. Since different industries produce different carbon emissions due to different structures, it is necessary to carry out detailed and specific exploration of various industries in China. Secondly, when studying the influencing factors, most literature did not consider the influence of technology, which is unreasonable. Therefore, factors such as technical efficiency and technological progress must be considered in the model, so as to examine its mechanism more precisely. Thirdly, when investigating the driving factors of carbon emissions, the decomposition results of most literature is basically consistent, such as carbon emission coefficient, energy intensity, energy mix, etc. Therefore, it is necessary to introduce new influencing factors to conduct more in-depth research on them.

## 3. Methodology and Data

### 3.1. Shephard Distance Function

Based on the PDA decomposition of environmental production technology, the decomposition model of the Shephard distance function is created by introducing energy input, economic output and carbon emission output. If energy, labor, and capital are the input factors, output value is the desired output, and carbon emissions are the undesired output, as described by the following equation:

(1) $$R^{t}=\left(E^{t},L^{t},K^{t},Y^{t},C^{t}\right),$$

indicating that (*E^t^*, *L^t^*, *K^t^*) can generate (*Y^t^*, *C^t^*).

In the above equation, $E^{t}\in R^{+}$ indicates energy input, $L^{t}\in R^{+}$ represents labor input, $K^{t}\in R^{+}$ is capital investment, $Y^{t}\in R^{+}$ represents desired output (or gross domestic production (GDP)), and $C^{t}\in R^{+}$ denotes the undesired output, i.e., carbon emissions. The Shephard distance function is introduced based on the production technology used in a given industry. The Shephard distance function of the *t*-period is expressed as:

(2) $$D_{e}^{t}\left(E^{t},L^{t},K^{t},Y^{t},C^{t}\right)=\sup\left\{\alpha :\left(E^{t}/\alpha ,L^{t},K^{t},Y^{t},C^{t}\right)\in S^{t}\right\}$$

(3) $$D_{y}^{t}\left(E^{t},L^{t},K^{t},Y^{t},C^{t}\right)=\inf\left\{\beta :\left(E^{t},L^{t},K^{t},Y^{t}/\beta ,C^{t}\right)\in S^{t}\right\}$$

(4) $$D_{c}^{t}\left(E^{t},L^{t},K^{t},Y^{t},C^{t}\right)=\sup\left\{\delta :\left(E^{t},L^{t},K^{t},Y^{t},C^{t}/\delta \right)\in S^{t}\right\}.$$

In this equation, $D_{e}^{t}(E^{t},L^{t},K^{t},Y^{t},C^{t}),D_{y}^{t}(E^{t},L^{t},K^{t},Y^{t},C^{t}),D_{c}^{t}(E^{t},L^{t},K^{t},Y^{t},C^{t})$ represent the optimal production front-edge distance, and $S^{t}$ indicates the possible set of production technologies. When $D_{e}^{t}\left(E^{t},L^{t},K^{t},Y^{t},C^{t}\right)\geq 1$, the smaller the value is, the closer the decision unit is to the probability boundary, and the higher the efficiency is. When $0\leq D_{y}^{t}\left(E^{t},L^{t},K^{t},Y^{t},C^{t}\right)\leq 1$, the larger the value is, the closer the decision unit is to the probability boundary, and the higher is the output efficiency. When $D_{c}^{t}\left(E^{t},L^{t},K^{t},Y^{t},C^{t}\right)\geq 1$, the smaller the value is, the closer the decision unit is to the probability boundary, and the higher is the efficiency. Once all the three distance functions reach a value of 1, the decision unit is at the forefront of production.

The intertemporal distance function, based on the definition of the Shephard distance function, was used in this study. Different periods are included in the same distance function, in which s indicates the base period of production technology and t represents the period in which the decision unit is located, as expressed in the following equations:

(5) $$D_{e}^{s}\left(E^{t},L^{t},K^{t},Y^{t},C^{t}\right)=\sup\left\{\alpha :\left(E^{t}/\alpha ,L^{t},K^{t},Y^{t},C^{t}\right)\in S^{t}\right\}$$

(6) $$D_{y}^{s}\left(E^{t},L^{t},K^{t},Y^{t},C^{t}\right)=\inf\left\{\beta :\left(E^{t},L^{t},K^{t},Y^{t}/\beta ,C^{t}\right)\in S^{t}\right\}$$

(7) $$D_{c}^{s}\left(E^{t},L^{t},K^{t},Y^{t},C^{t}\right)=\sup\left\{\delta :\left(E^{t},L^{t},K^{t},Y^{t},C^{t}/\delta \right)\in S^{t}\right\}.$$

To solve Equations (5)–(7), the scale returns are assumed to be constant and the following equations were used to make the calculations:

(8) $$D_{e}^{s}{\left(E^{t},L^{t},K^{t},Y^{t},C^{t}\right)}^{-1}=\min\alpha \;s.t\{\begin{array}{c} \lambda E^{s}\leq \alpha E^{t}\\ \lambda L^{s}\leq L^{t}\\ \lambda K^{s}\leq K^{t}\\ \lambda Y^{s}\geq Y^{t}\\ \lambda C^{s}=C^{t}\\ \lambda \geq 0,s,t\in \left\{T,T+1\right\} \end{array}$$

(9) $$D_{c}^{s}{\left(E^{t},L^{t},K^{t},Y^{t},C^{t}\right)}^{-1}=\min\delta \;t\{\begin{array}{c} \lambda E^{s}\leq E^{t}\\ \lambda L^{s}\leq L^{t}\\ \lambda K^{s}\leq K^{t}\\ \lambda Y^{s}\geq Y^{t}\\ \lambda C^{s}=\delta C^{t}\\ \lambda \geq 0,s,t\in \left\{T,T+1\right\} \end{array}$$

(10) $$D_{y}^{s}{\left(E^{t},L^{t},K^{t},Y^{t},C^{t}\right)}^{-1}=\max\beta \;t\{\begin{array}{c} \lambda E^{s}\leq E^{t}\\ \lambda L^{s}\leq L^{t}\\ \lambda K^{s}\leq K^{t}\\ \lambda Y^{s}\geq \beta Y^{t}\\ \lambda C^{s}=C^{t}\\ \lambda \geq 0,s,t\in \left\{T,T+1\right\} \end{array}.$$

### 3.2. PDA Production Decomposition Model

Kaya’s identity was first proposed by Kaya [18]. This study extended the Kaya identity, and employed it to preliminarily decompose CO_2_ into:

(11) $$C=\sum_{j}\frac{C_{j}}{E_{j}}\times \frac{E_{j}}{E}\times \frac{E}{Y}\times Y.$$

In the above equation, *C* is the total amount of carbon emissions, *C_j_* is CO_2_ produced from the *j* energy source, *E_j_* is the total consumption of the *j* energy, *E* is the total energy consumption, and *Y* is the added value of industry. In this study, the above equation was expanded again according to the decomposition method of Zhou and Ang [38]. To avoid subjective influence, the correlation efficiency of the distance function was determined after geometrically averaging values of the *t*-period and *t* + 1 period. The specific decomposition model is as follows.

The decomposition model of carbon emissions in *t* period:

(12) $$\begin{array}{l} C^{t}=\sum_{j}\frac{C_{j}^{t}}{{\left[D_{C}^{t}(K^{t},L^{t},E^{t},Y^{t},C^{t})\times D_{C}^{t+1}(K^{t},L^{t},E^{t},Y^{t},C^{t})\right]}^{\frac{1}{2}}}\times \frac{1}{E_{j}^{t}}\times \\ \frac{E_{j}^{t}}{E^{t}}\times \frac{E^{t}}{{\left[D_{E}^{t}(K^{t},L^{t},E^{t},Y^{t},C^{t})\times D_{E}^{t+1}(K^{t},L^{t},E^{t},Y^{t},C^{t})\right]}^{\frac{1}{2}}}\times \frac{1}{Y^{t}}\\ \times Y^{t}\times {\left[D_{Y}^{t}(K^{t},L^{t},E^{t},Y^{t},C^{t})\times D_{Y}^{t+1}(K^{t},L^{t},E^{t},Y^{t},C^{t})\right]}^{\frac{1}{2}}\times \\ D_{C}^{t}(K^{t},L^{t},E^{t},Y^{t},C^{t})\times {\left[\frac{D_{C}^{t+1}(K^{t},L^{t},E^{t},Y^{t},C^{t})}{D_{C}^{t}(K^{t},L^{t},E^{t},Y^{t},C^{t})}\right]}^{{}^{\frac{1}{2}}}\times \\ D_{E}^{t}(K^{t},L^{t},E^{t},Y^{t},C^{t})\times {\left[\frac{D_{E}^{t+1}(K^{t},L^{t},E^{t},Y^{t},C^{t})}{D_{E}^{t}(K^{t},L^{t},E^{t},Y^{t},C^{t}}\right]}^{{}^{\frac{1}{2}}}\\ \times \frac{1}{D_{Y}^{t}(K^{t},L^{t},E^{t},Y^{t},C^{t})}\times {\left[\frac{D_{Y}^{t}(K^{t},L^{t},E^{t},Y^{t},C^{t})}{D_{Y}^{t+1}(K^{t},L^{t},E^{t},Y^{t},C^{t})}\right]}^{{}^{\frac{1}{2}}} \end{array}.$$

The decomposition model of carbon emissions in *t* + 1 period:

(13) $$\begin{array}{l} C^{t+1}=\sum_{j}\frac{C_{j}^{t+1}}{{\left[D_{C}^{t}(K^{t+1},L^{t+1},E^{t+1},Y^{t+1},C^{t+1})\times D_{C}^{t+1}(K^{t+1},L^{t+1},E^{t+1},Y^{t+1},C^{t+1})\right]}^{\frac{1}{2}}}\times \frac{1}{E_{j}^{t+1}}\times \\ \frac{E_{j}^{t+1}}{E^{t+1}}\times \frac{E^{t+1}}{{\left[D_{E}^{t}(K^{t+1},L^{t+1},E^{t+1},Y^{t+1},C^{t+1})\times D_{E}^{t+1}(K^{t+1},L^{t+1},E^{t+1},Y^{t+1},C^{t+1})\right]}^{\frac{1}{2}}}\times \frac{1}{Y^{t+1}}\\ \times Y^{t+1}\times {\left[D_{Y}^{t}(K^{t+1},L^{t+1},E^{t+1},Y^{t+1},C^{t+1})\times D_{Y}^{t+1}(K^{t+1},L^{t+1},E^{t+1},Y^{t+1},C^{t+1})\right]}^{\frac{1}{2}}\times \\ D_{C}^{t+1}(K^{t+1},L^{t+1},E^{t+1},Y^{t+1},C^{t+1})\times {\left[\frac{D_{C}^{t}(K^{t+1},L^{t+1},E^{t+1},Y^{t+1},C^{t+1})}{D_{C}^{t+1}(K^{t+1},L^{t+1},E^{t+1},Y^{t+1},C^{t+1})}\right]}^{{}^{\frac{1}{2}}}\times \\ D_{E}^{t+1}(K^{t+1},L^{t+1},E^{t+1},Y^{t+1},C^{t+1})\times {\left[\frac{D_{E}^{t}(K^{t+1},L^{t+1},E^{t+1},Y^{t+1},C^{t+1})}{D_{E}^{t+1}(K^{t+1},L^{t+1},E^{t+1},Y^{t+1},C^{t+1})}\right]}^{{}^{\frac{1}{2}}}\\ \times \frac{1}{D_{Y}^{t+1}(K^{t+1},L^{t+1},E^{t+1},Y^{t+1},C^{t+1})}\times {\left[\frac{D_{Y}^{t+1}(K^{t+1},L^{t+1},E^{t+1},Y^{t+1},C^{t+1})}{D_{Y}^{t}(K^{t+1},L^{t+1},E^{t+1},Y^{t+1},C^{t+1})}\right]}^{{}^{\frac{1}{2}}} \end{array}.$$

Equations (12) and (13) can be briefly expressed as:

(14) $$\begin{array}{ll} C^{S}= & \sum_{J}PCECH_{J}^{S}\times PEMCH_{J}^{S}\times PEICH\times PGDPCH\times CETECH\times CETCH\times EUTECH\\ & \times EUTCH\times GDPTECH\times GDPTCH\;\;\;S\in \left\{0,T\right\} \end{array}.$$

Taking Equation (12) as an example, Part 1 indicates the potential energy carbon intensity of a sub-sector, and is recorded as PCECH:

(15) $$PCECH_{J}^{t}=\frac{C_{j}^{t}}{{\left[D_{C}^{t}(K^{t},L^{t},E^{t},Y^{t},C^{t})\times D_{C}^{t+1}(K^{t},L^{t},E^{t},Y^{t},C^{t})\right]}^{\frac{1}{2}}}\times \frac{1}{E_{j}^{t}}.$$

Part 2 is the energy mix of different industries that shows the proportion of a certain energy type in the total energy consumption, and is represented as PEMCH:

(16) $$PEMCH_{J}^{t}=E_{j}^{t}/E^{t}.$$

Part 3 is the potential energy intensity factor of the industrial sector, and is recorded as PEICH, i.e.,

(17) $$PEICH=\frac{E^{t}}{{\left[D_{E}^{t}(K^{t},L^{t},E^{t},Y^{t},C^{t})\times D_{E}^{t+1}(K^{t},L^{t},E^{t},Y^{t},C^{t})\right]}^{\frac{1}{2}}}\times \frac{1}{Y^{t}},$$

which refers to the energy intensity adjusted by energy utilization efficiency. 

Part 4 is the potential GDP factor in the industrial sector, and is denoted as PGDPCH, i.e.,

(18) $$PGDPCH=Y^{t}\times {\left[D_{Y}^{t}(K^{t},L^{t},E^{t},Y^{t},C^{t})\times D_{Y}^{t+1}(K^{t},L^{t},E^{t},Y^{t},C^{t})\right]}^{\frac{1}{2}},$$

which refers to the actual output after adjustment of output efficiency.

Based on the definition of the Malmquist index, Parts 5 and 6 are, respectively, the technical efficiency of carbon emissions and technological progress of carbon emissions, which are recorded as CETECH and CETCH, respectively:

(19) $$CETECH=D_{C}^{t}(K^{t},L^{t},E^{t},Y^{t},C^{t})$$

(20) $$CETCH={\left[\frac{D_{C}^{t+1}(K^{t},L^{t},E^{t},Y^{t},C^{t})}{D_{C}^{t}(K^{t},L^{t},E^{t},Y^{t},C^{t})}\right]}^{{}^{\frac{1}{2}}}.$$

Parts 7 and 8 are changes of technological efficiency and progress of energy use, respectively, which are represented as EUTECH and EUTCH, respectively:

(21) $$EUTECH=D_{E}^{t}(K^{t},L^{t},E^{t},Y^{t},C^{t})$$

(22) $$EUTCH={\left[\frac{D_{E}^{t+1}(K^{t},L^{t},E^{t},Y^{t},C^{t})}{D_{E}^{t}(K^{t},L^{t},E^{t},Y^{t},C^{t}}\right]}^{{}^{\frac{1}{2}}}.$$

Parts 9 and 10 are, respectively, changes of technological efficiency and progress of economic output, which are recorded as GDPTECH and GDPTCH, respectively:

(23) $$GDPTECH=\frac{1}{D_{Y}^{t}(K^{t},L^{t},E^{t},Y^{t},C^{t})}$$

(24) $$GDPTCH={\left[\frac{D_{Y}^{t}(K^{t},L^{t},E^{t},Y^{t},C^{t})}{D_{Y}^{t+1}(K^{t},L^{t},E^{t},Y^{t},C^{t}}\right]}^{{}^{\frac{1}{2}}}.$$

Addition and multiplication are the two mathematical operations in the solution process for LMDI. The additive form is reported in many literature. However, to better evaluate the industrial carbon emissions problem, multiplication was used to solve the LMDI in this study and quantitatively analyze the driving factors. The specific equation is as follows:

(25) $$D=C^{t+1}/C^{t}=D_{\left(PCECH\right)}\times D_{\left(PEMCH\right)}\times D_{\left(PEICH\right)}\times D_{\left(PGDPCH\right)}\times D_{\left(CETECH\right)}\times D_{\left(CETCH\right)}\;\times D_{\left(EUTECH\right)}\times D_{\left(EUTCH\right)}\times D_{\left(GDPTECH\right)}\times D_{\left(GDPTCH\right)}.$$

The equation for calculating the driving factors is as follows:

(26) $$D_{\left(PCECH\right)}=\exp\left\{\sum_{j}\frac{\left(C_{J}^{t+1}-C_{J}^{t}\right)/\left(\ln C_{J}^{t+1}-\ln C_{J}^{t}\right)}{\left(C^{t+1}-C^{t}\right)/\left(\ln C^{t+1}-\ln C^{t}\right)}\times \ln\left(\frac{PCECH_{J}^{t+1}}{PCECH_{J}^{t}}\right)\right\}$$

(27) $$D_{\left(PEMCH\right)}=\exp\left\{\sum_{j}\frac{\left(C_{J}^{t+1}-C_{J}^{t}\right)/\left(\ln C_{J}^{t+1}-\ln C_{J}^{t}\right)}{\left(C^{t+1}-C^{t}\right)/\left(\ln C^{t+1}-\ln C^{t}\right)}\times \ln\left(\frac{PEMCH_{J}^{t+1}}{PEMCH_{J}^{t}}\right)\right\}$$

(28) $$D_{\left(PEICH\right)}=\exp\left\{\sum_{j}\frac{\left(C_{J}^{t+1}-C_{J}^{t}\right)/\left(\ln C_{J}^{t+1}-\ln C_{J}^{t}\right)}{\left(C^{t+1}-C^{t}\right)/\left(\ln C^{t+1}-\ln C^{t}\right)}\times \ln\left(\frac{PEICH^{t+1}}{PEICH^{{}^{t}}}\right)\right\}$$

(29) $$D_{\left(PGDPCH\right)}=\exp\left\{\sum_{j}\frac{\left(C_{J}^{t+1}-C_{J}^{t}\right)/\left(\ln C_{J}^{t+1}-\ln C_{J}^{t}\right)}{\left(C^{t+1}-C^{t}\right)/\left(\ln C^{t+1}-\ln C^{t}\right)}\times \ln\left(\frac{PGDPCH^{t+1}}{PGDPCH^{{}^{t}}}\right)\right\}$$

(30) $$D_{\left(CETECH\right)}=\exp\left\{\sum_{j}\frac{\left(C_{J}^{t+1}-C_{J}^{t}\right)/\left(\ln C_{J}^{t+1}-\ln C_{J}^{t}\right)}{\left(C^{t+1}-C^{t}\right)/\left(\ln C^{t+1}-\ln C^{t}\right)}\times \ln\left(\frac{CETECH^{t+1}}{CETECH^{{}^{t}}}\right)\right\}$$

(31) $$D_{\left(CETCH\right)}=\exp\left\{\sum_{j}\frac{\left(C_{J}^{t+1}-C_{J}^{t}\right)/\left(\ln C_{J}^{t+1}-\ln C_{J}^{t}\right)}{\left(C^{t+1}-C^{t}\right)/\left(\ln C^{t+1}-\ln C^{t}\right)}\times \ln\left(\frac{CETCH^{t+1}}{CETCH^{{}^{t}}}\right)\right\}$$

(32) $$D_{\left(EUTECH\right)}=\exp\left\{\sum_{j}\frac{\left(C_{J}^{t+1}-C_{J}^{t}\right)/\left(\ln C_{J}^{t+1}-\ln C_{J}^{t}\right)}{\left(C^{t+1}-C^{t}\right)/\left(\ln C^{t+1}-\ln C^{t}\right)}\times \ln\left(\frac{EUTECH^{t+1}}{EUTECH^{{}^{t}}}\right)\right\}$$

(33) $$D_{\left(EUTCH\right)}=\exp\left\{\sum_{j}\frac{\left(C_{J}^{t+1}-C_{J}^{t}\right)/\left(\ln C_{J}^{t+1}-\ln C_{J}^{t}\right)}{\left(C^{t+1}-C^{t}\right)/\left(\ln C^{t+1}-\ln C^{t}\right)}\times \ln\left(\frac{EUTCH^{t+1}}{EUTCH^{{}^{t}}}\right)\right\}$$

(34) $$D_{\left(GDPTECH\right)}=\exp\left\{\sum_{j}\frac{\left(C_{J}^{t+1}-C_{J}^{t}\right)/\left(\ln C_{J}^{t+1}-\ln C_{J}^{t}\right)}{\left(C^{t+1}-C^{t}\right)/\left(\ln C^{t+1}-\ln C^{t}\right)}\times \ln\left(\frac{GDPTECH^{t+1}}{GDPTECH^{{}^{t}}}\right)\right\}$$

(35) $$D_{\left(GDPTCH\right)}=\exp\left\{\sum_{j}\frac{\left(C_{J}^{t+1}-C_{J}^{t}\right)/\left(\ln C_{J}^{t+1}-\ln C_{J}^{t}\right)}{\left(C^{t+1}-C^{t}\right)/\left(\ln C^{t+1}-\ln C^{t}\right)}\times \ln\left(\frac{GDPTCH^{t+1}}{GDPTCH^{{}^{t}}}\right)\right\}.$$

In the solution process of the decomposition model, the consumption amount might be zero for certain types of energy within certain industries in certain years, which is not conducive to calculating the contribution of the driving factors. Under such conditions, the method used by Ang et al. [42] to address zero values was adopted. This approach replaces the zero value with a very small positive number (such as 10–10 or 10–20) to eliminate the large errors.

### 3.3. Data

Except for carbon emissions, the data used in the study were sourced from the *Chinese Statistical Yearbook* [45], *China Energy Statistical Yearbook* [46], *China Industrial Statistical Yearbook* [47], *Statistical Yearbook of the Chinese Investment in Fixed Assets* [48], *China Population and Employment Statistics Yearbook* [49], *China Price Statistics Yearbook* [50] and *China Input‒Output Table* [51]. All data are for the period 2003 to 2015. The data description is shown in Table 1.

Tian’s [52] partial calculation results and perpetual inventory method were employed in calculating the data on capital stock and the depreciation rate was calculated based on the depreciation data of fixed assets stated in the *China Input‒Output Table*.

The emissions of CO_2_ from different industries were calculated by selecting the coal, aggregated oil products, and natural gas given in the *China Energy Statistical Yearbook*, using the following equation:

(36) $$C=\sum_{j}E_{j}\times \alpha_{j}\times \beta_{j}\times \frac{44}{12},$$

where $E_{j}$ refers to the consumption of energy *j*; $\alpha_{j}$ refers to the standard coal coefficient converted from energy *j*; $\beta_{j}$ represents the carbon emission coefficient of energy *j*; the constant 44/12 is the mass conversion coefficient of the CO_2_ molecule oxidized by carbon, and *C* indicates the CO_2_ emissions from the terminal energy consumption of the industry.

Table 2 presents the division of industries. Some industries are an aggregate of similar industries. Industry (4) represents the ferrous and non-ferrous metal mining, and industry (6) is a combination of four industries, i.e., agricultural and sideline products, food manufacturing, beverages, and tobacco products. Industry (7) is a combination of three industries, i.e., textiles and apparels, footwear, and leather and fur. Industry (8) includes wood processing and related product manufacturing, such as furniture. Industry (9) is a combination of three industries, i.e., paper-making and related products, printing, and cultural and educational supplies manufacturing. Industry (11) is a combination of four industries, i.e., chemical raw materials and chemical products, medicine manufacturing, rubber and plastic products, and chemical fiber manufacturing. Industry (13) includes ferrous and non-ferrous metal smelting and rolling processing. Industry (15) refers to general and special equipment manufacturing.

## 4. Results and Discussion

### 4.1. Analysis of Carbon Emissions from Different Industries

**Figure 1:**
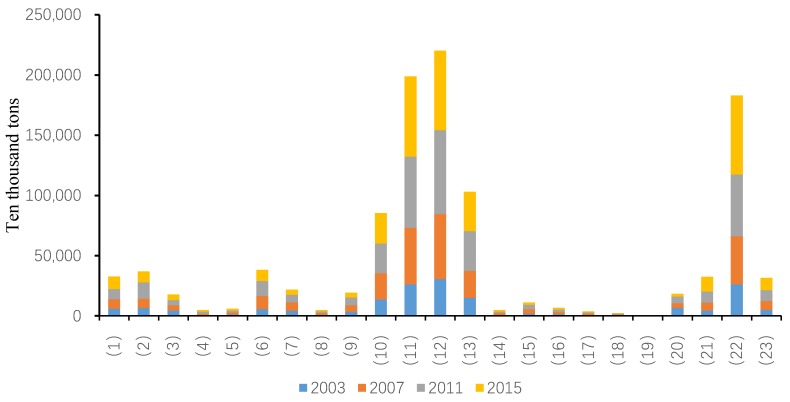
Energy-related CO_2_ emissions in each industry.

Data show that CO_2_ emissions of different industries in China during the study period are significantly different. As is shown in Figure 1, CO_2_ emissions were especially high in the petroleum processing industry (10), the chemical industry (11), the non-metallic mineral products industry (12), and the transportation, warehousing and postal industries (22). Comparatively, the CO_2_ emissions are relatively lower in the metal mining industry (4), the wood processing, wood products and furniture manufacturing industry (8), the metal products manufacturing industry (14), the electrical machinery and equipment manufacturing industry (17), the computer manufacturing industry (18), and the instrumentation manufacturing industry (19). The CO_2_ emissions of the remaining industries are relatively small and remain constant at a moderate level. According to the above analysis, except for the transportation industry (22), all industries with particularly high emissions are classified as “secondary” industry. Notably, the six industries with relatively low CO_2_ emissions also belong to the secondary industry, thus proving that not all of industries within the secondary industry discharge high carbon emissions and cause high levels of pollution. Furthermore, there are certain low-carbon industries in China as well as high-emission industries. Therefore, when measures are proposed or enacted to reduce and curb carbon emissions, attention needs to be paid to the differences among industries. Nevertheless, the data definitively specify that most carbon dioxide emissions are generated from the secondary industry. Notably, the transportation industry (22) also has a particularly high amount of CO_2_ emissions, illustrating that the current tertiary industry is not completely low-carbon. Hence it is necessary to strictly reduce and control carbon emissions and develop suitable carbon emission reduction measures for the tertiary industry as well as for the secondary industry.

**Figure 2:**
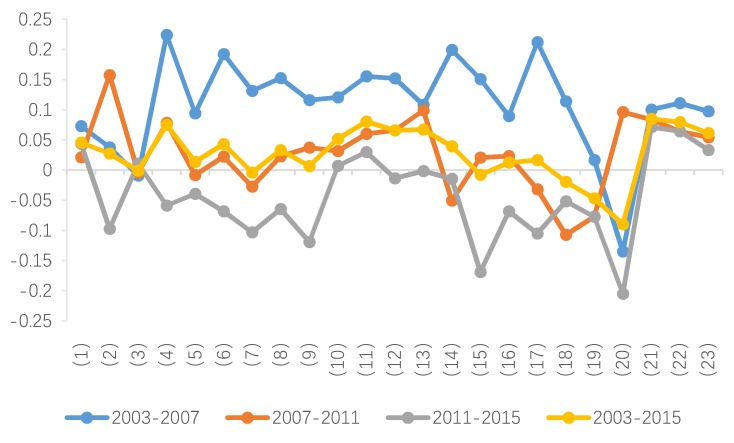
Growth rate of energy-related CO_2_ emissions in each industry.

As shown in Figure 2, in view of the average annual growth rate of carbon emissions from 2003 to 2015, six industries register negative growth rates of carbon emissions. These industries are oil and natural gas extraction (3), the textile industry and related products (7), general and special equipment manufacturing (15), computer manufacturing (18), instrumentation manufacturing (19), and power and heat production and supply (20). Carbon emissions in industry (3) do not show over-fluctuation during the study period. Industries (7), (15), (18) and (19) initially present an upward trend in carbon emission growth but the trend eventually turn downward. The carbon emission growth of industry (20) initially decreases tremendously, but then quickly increases and then slowly decreases, exhibiting a negative overall growth rate.

In 2003–2015, among the industries with positive annual growth rate of carbon emissions, the construction industry (21) ranks first, followed by the chemical industry (11) and the transportation industry (22). The growth of carbon emissions from these three industries continually increases over the study period. Other industries that exhibit a continued increase of carbon emissions are the wholesale and retail, accommodation and catering industry (23), the petroleum processing, coking and nuclear fuel processing industry (10), and the agriculture, forestry, animal husbandry and fishery industry (1). For the remaining 11 industries at specific stages of the study period, the growth rates of carbon emissions tend to be sometimes positive or negative, but are not always increasing. The 11 industries are metal mining (4), metal smelting and rolling processing (13), non-metallic mineral products manufacturing (12), food manufacturing and tobacco processing (6), metal products (14), wood processing and products, furniture manufacturing (8), coal mining and washing (2), electrical machinery and equipment manufacturing (17), non-metal mining and other mining (5), transportation equipment manufacturing (16) and paper-making, printing, culture and education (9). Overall, the growth rate of carbon emissions in these 11 industries decreases during 2003–2015.

The average growth rates of carbon emissions in most industries during 2003–2015 increases, which lead to the continuous increase of total CO_2_ emissions. However, the growth rate of carbon emissions in most industries increases from 2003 to 2007, followed by slower growth from 2007 to 2011 and decreasing growth from 2011 to 2015. Statistical analysis of carbon emissions data from selected industries suggest that the total amount of carbon emissions continues to increase throughout the study period, yet the specific changes vary among different industries.

### 4.2. Driving Factors Analysis

**Figure 3:**
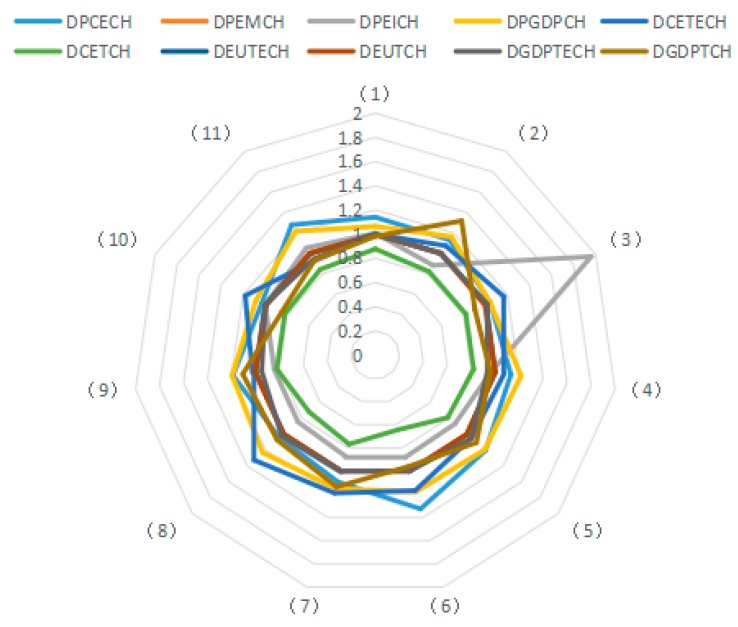
Results of factor decomposition for carbon emissions during 2003–2015.

**Figure 4:**
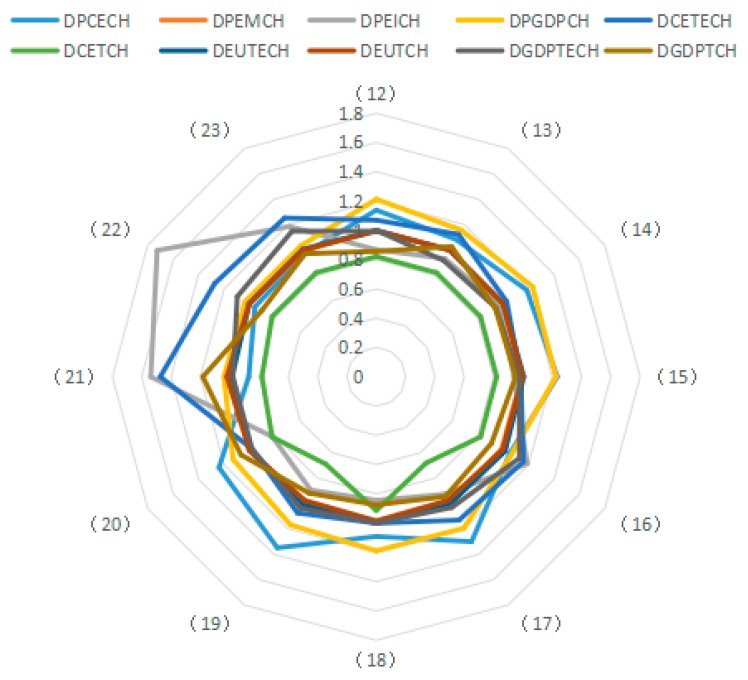
Results of factor decomposition for carbon emissions during 2003–2015.

**Figure 5:**
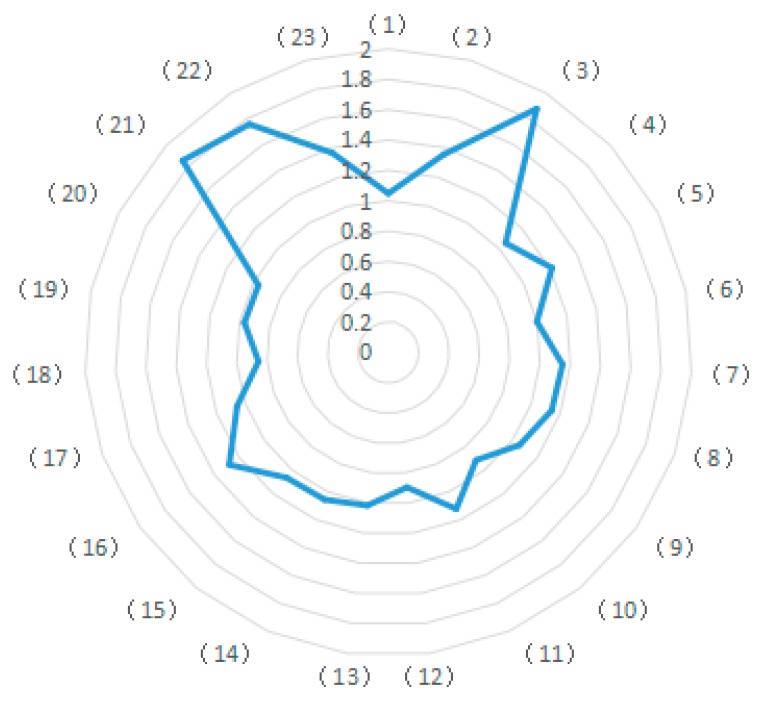
Change rate of carbon emissions in each industry during 2003–2015.

Using the decomposition formula composed of the Shephard distance function, PDA production decomposition analysis and LMDI decomposition, the influence of different driving factors on carbon emissions from industry was examined. Results illustrated in Figure 3, Figure 4 and Figure 5 show the average of the annual decomposition of the factors of carbon emissions from 23 industries during 2003–2015. In these figures, the values was >1 (<1 or =1) indicate that a factor increases (decreases or does not contribute to) CO_2_ emissions.

(1) Potential Energy Carbon Intensity

**Figure 6:**
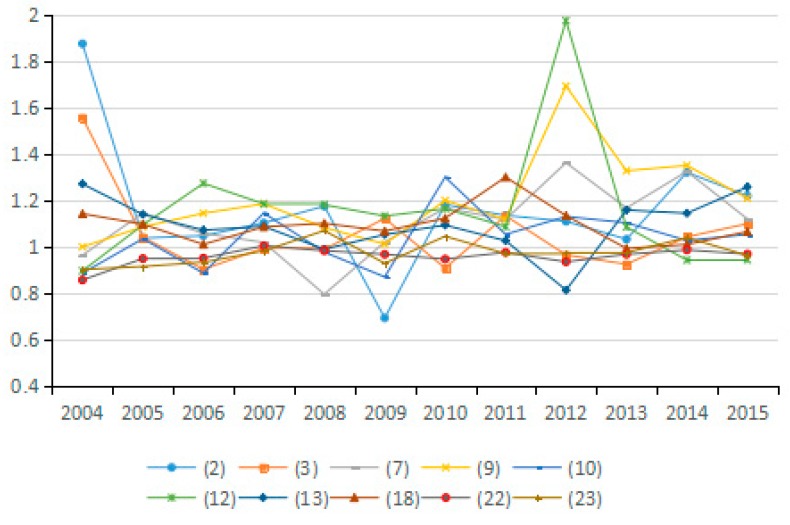
Changes in potential energy carbon intensity (sub-industry).

**Figure 7:**
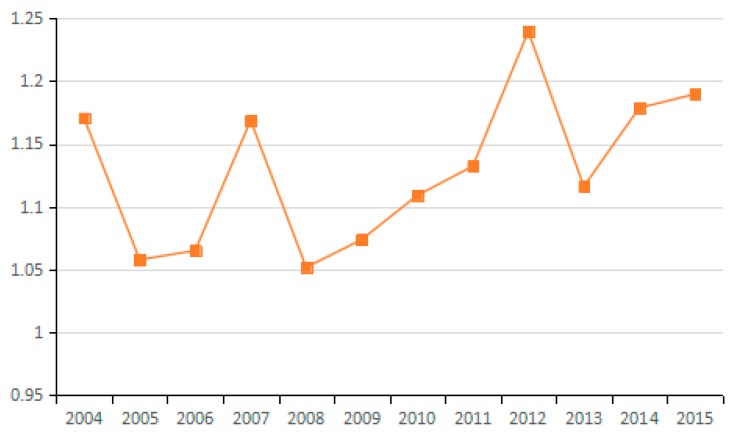
Changes in potential energy carbon intensity (industry average change).

Based on the factor decomposition results shown in Figure 3, Figure 4 and Figure 5, 10 representative industries were selected to illustrate the direction of change in potential energy carbon intensity factors in different industries (see Figure 6). Figure 7 reflects the average value of potential carbon intensity changes of all industries. Potential energy carbon intensity is a factor that considers the efficiency of carbon emission technology, that is to say, the low efficiency of controlling CO_2_ emissions will lead to the observed carbon emission factor being greater than that of the original carbon emission factor. The average change in the value of potential energy carbon intensity factors for each of the 23 industries from 2003 to 2015 is mostly >1, which indicates that the reduction of carbon emissions is hindered and that China’s energy carbon intensity does not present much improvement. The reason for this result is that among the three fossil energy sources, coal has a large emission coefficient, and coal still accounts for as much as 65% of China’s total energy consumption. The large consumption of coal will continue to increase carbon emissions through the influence of the potential energy carbon intensity factor. This conclusion is consistent with Sun et al., although the subject of the study is different [29]. However, the potential energy carbon intensity factor of industries (21), (22), and (23) plays a role in reducing carbon emissions.

(2) Structural Factors of Energy Consumption

**Figure 8:**
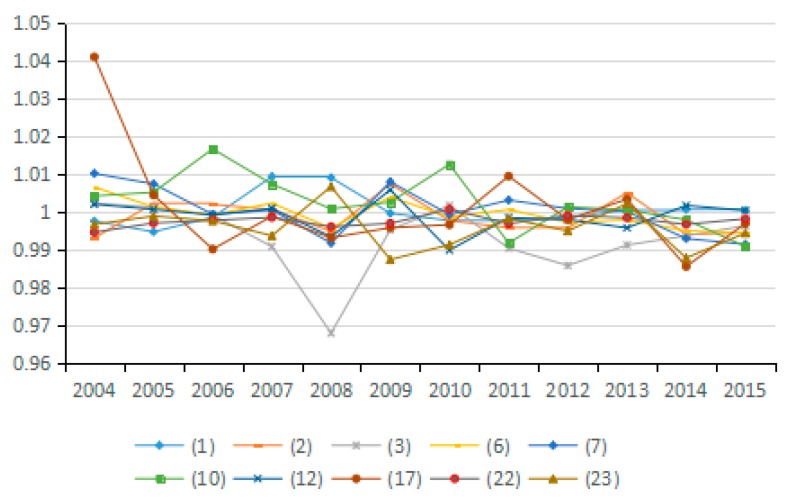
Changes in energy mix (sub-industry).

**Figure 9:**
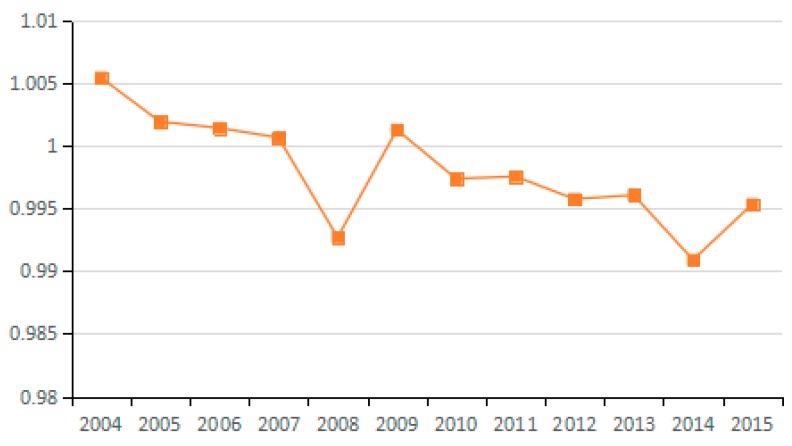
Changes in energy mix (industry average change).

According to the average values for the energy consumption structure factors of various industries from 2003 to 2015, most of them play a role in reducing carbon emissions (see Figure 8 and Figure 9). However, only the energy consumption structural changes in industries (1), (5), (7), (10), (11), (13), and (17) obviously inhibit the reduction of carbon emissions. These results indicate that coal use is effectively controlled during the development of most industries, thus helping to reduce CO_2_ emissions.

Undoubtedly, the energy mix factor can reduce carbon emissions, but the decomposition results specify that the emission reduction effect of this factor is not obvious. For all 23 sampled industries, the energy consumption structure factors have values close to 1 without obvious differences among industries. In studying the multi-regional carbon emission factors, Ma et al. also found that the energy structure has a small reduction effect [53]. This result indicates that the energy consumption structure of most industries in China has not improved significantly. Fundamentally, this is because the current energy consumption is still dominated by coal. These results further demonstrate that China’s clean energy initiative has not been fully popularized and coal still dominates the energy consumption in Chinese industry. Thus, there is still a huge potential for reducing carbon emissions via adjusting and optimizing the energy consumption structure.

(3) Potential Energy Intensity

**Figure 10:**
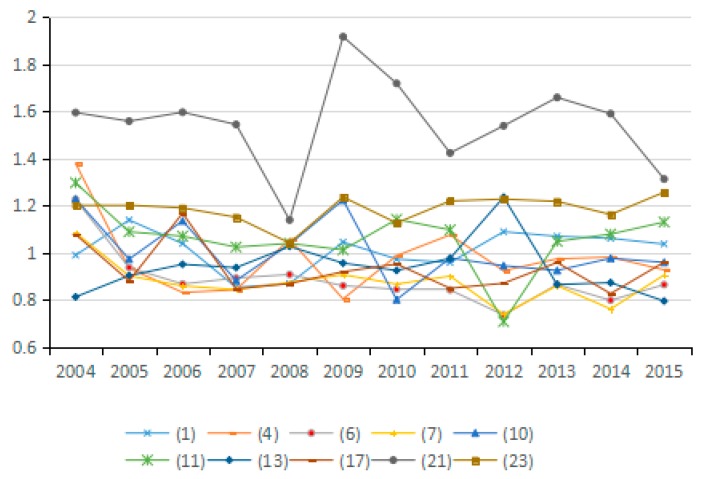
Changes in potential energy intensity (sub-industry).

**Figure 11:**
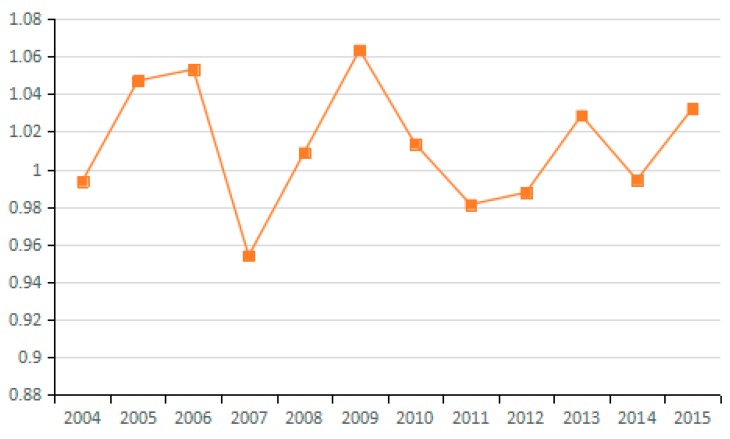
Changes in potential energy intensity (industry average change).

In general, actual energy intensity reflects the actual energy use efficiency. However, the potential energy intensity refers to energy intensity that would result from adjusting the energy input efficiency. During the research period, the improvement of energy input efficiency increases the potential energy intensity, thus increasing the influence of energy intensity on carbon emissions. The average value of changes in the potential energy intensity factor for most of the 23 industries from 2003 to 2015 is <1. Thus, this factor has a positive effect on carbon emission reduction (see Figure 10 and Figure 11), and its effect on emission reduction is consistent with that of energy intensity. Wang et al. explored China’s carbon emissions and also found that potential energy carbon intensity factors have an inhibitory effect on carbon emissions [54].

The energy intensity factor is indicated by the ratio of energy consumption to industrial GDP. However, the potential energy intensity factor is adjusted via energy efficiency. Therefore, for industries with a potential energy intensity factor of less than 1, the growth rate of industrial GDP exceeds that of the industrial energy use, thus contributing to the reduction of carbon emissions. Conversely, for industries in which the change in the value of the potential energy intensity factor exceeds 1, the growth rate of industrial GPD is lower than that of energy consumption, which results in increased CO_2_ emissions. In China, the contribution of the potential energy intensity factor to emission reduction is tremendous. Hence, efforts should be made to enable potential energy intensity to play a positive role in reducing carbon emissions, such as by formulating industry-specific measures for realizing energy conservation and carbon emission reduction in a better and faster way.

(4) Potential GDP Factor

The potential GDP factor shows the influence of adjusted actual output by using the GDP output efficiency. If the output efficiency is increased, the changes in potential GDP are greater than those of the actual GDP, resulting in an amplified influence on changes in CO_2_ emissions. The change in the average value of the potential GDP factor for the 23 industries from 2003 to 2015 exceeds 1. Therefore, this factor is a main influence that increases carbon emissions.

**Figure 12:**
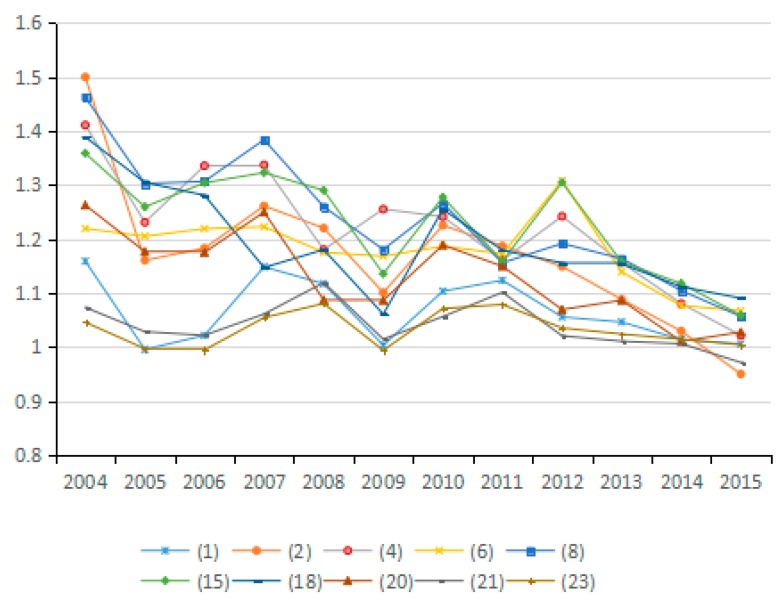
Changes in potential GDP (sub-industry).

**Figure 13:**
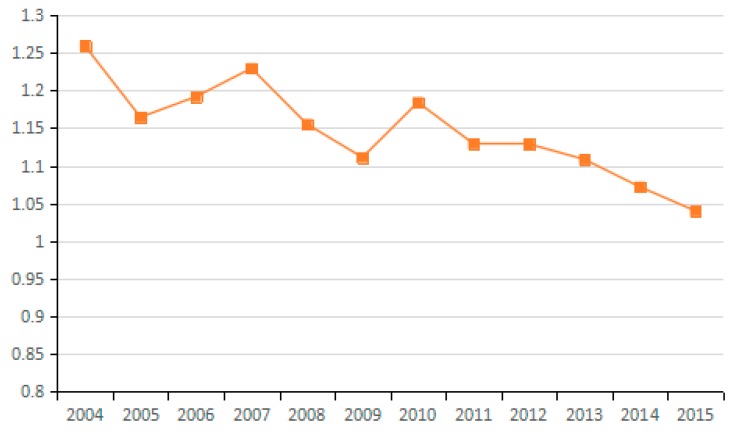
Changes in potential GDP (industry average change).

Whether considering the carbon emissions of individual industries or of China in general, the potential GDP factor not only determines the direction of carbon emission changes, but also to a large extent determines the scale of carbon emissions (see Figure 12 and Figure 13). The effect is consistent with the influence of GDP. This conclusion is consistent with Wang et al. They also found that potential GDP has a positive effect on carbon emissions [54]. Because China now is at the stage of accelerated development of industrialization and urbanization, the key task is to address the relationship between economic development and carbon emission reduction, thus achieving sustainable development.

(5) Technical Efficiency Factor of Carbon Emissions

**Figure 14:**
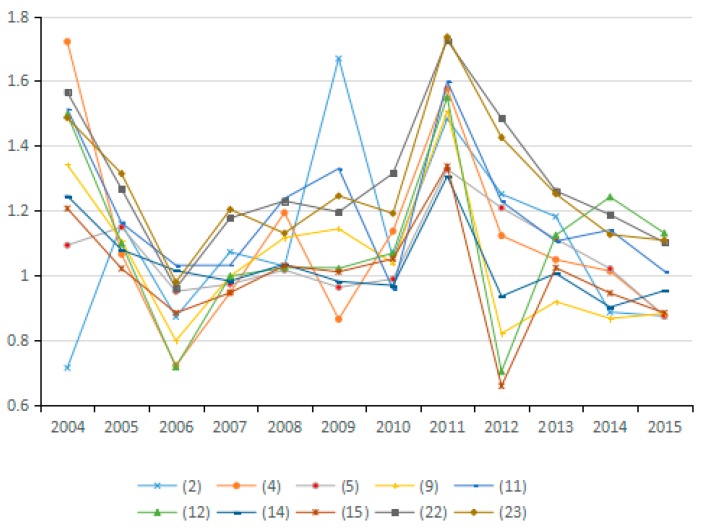
Changes in technical efficiency of carbon emissions (sub-industry).

**Figure 15:**
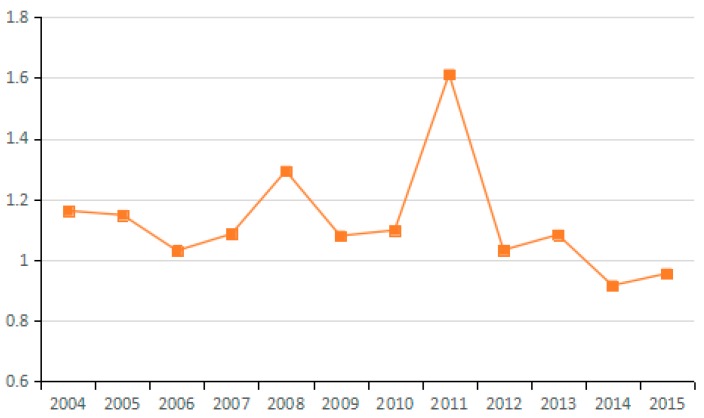
Changes in technical efficiency of carbon emissions (average change of industry).

Based on the Shephard distance function, the technical efficiency of carbon emissions indicates the possibility of reducing carbon emissions while other conditions remain unchanged. The values of technical efficiency factors of carbon emissions for most of the 23 industries (except industries (11), (15) and (20)) exceed 1 (see Figure 14). Thus, technical efficiency does not reduce carbon emissions, but on the contrary, increases the emissions. Seen from the overall perspective of the 23 industries, the technical efficiency levels of carbon emissions in 2003–2015 increase (see Figure 15). Theoretically, the improvement of efficiency is conducive to decreasing carbon emissions. However, the empirical evidence in this study specifies that technical efficiency improvements do not exert corresponding influences on carbon emissions in the sample period, which proves, to some extent, that China’s carbon emission technical efficiency level has not improved dramatically.

(6) Technological Advancement Factor of Carbon Emissions

**Figure 16:**
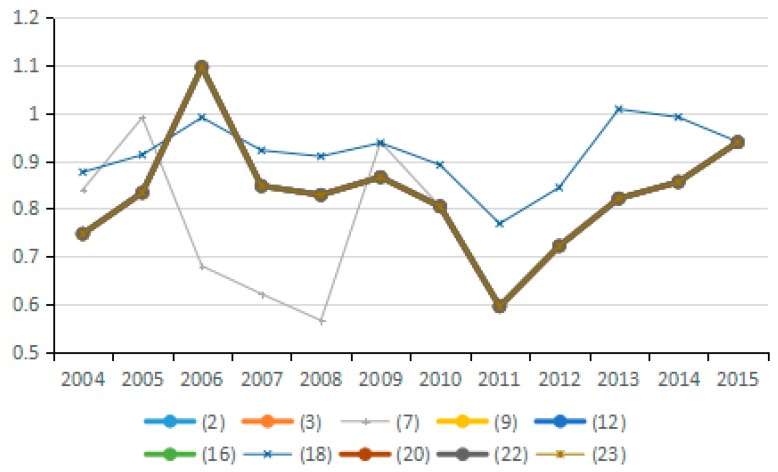
Changes in technical progress of carbon emissions (sub-industry).

**Figure 17:**
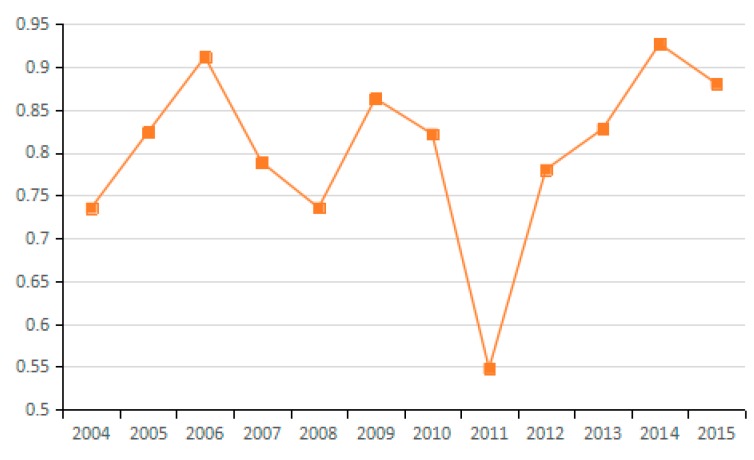
Changes in technical progress of carbon emissions (industry average change).

Decomposed by the Malmquist index, the technological advancement factor of carbon emissions is used to evaluate the technological progress of the decision-making unit. Figure 16 shows the results of decomposition and the direction of change in the technological advances of carbon emissions from 10 representative industries. Figure 17 presents the average changes in technological progress of the 23 industries. By analyzing the average values for technological progress of the 23 industries from 2003 to 2015, the technological progress in carbon emissions is shown to remain relatively constant at a value of 0.8212, indicating that the advancement of carbon emission technologies in the different industries is the same. This is mainly due to the fact that the general and special equipment manufacturing industry (15) shapes a frontier through the origin, which indicates the same proportion of technological progress in all industries. The technological progress of carbon emissions is generally <1 for the 23 industries, which means that technological progress plays a positive role in reducing carbon emissions, and there is a technological progress effect on carbon emissions from the industries.

(7) Energy Technology Efficiency Factor

**Figure 18:**
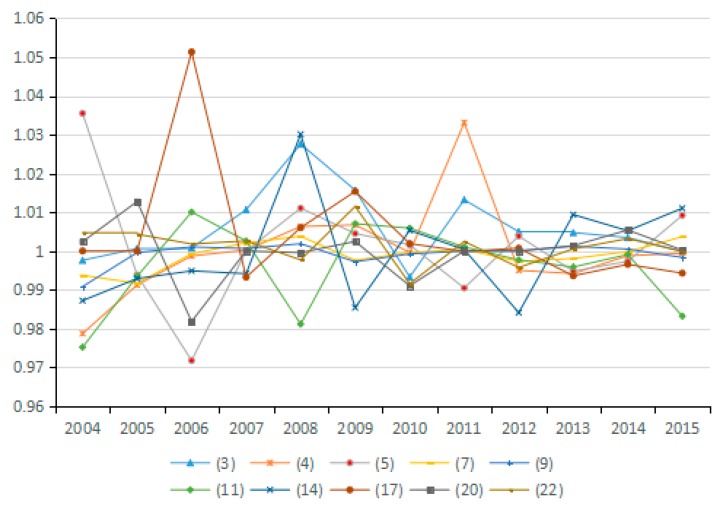
Changes in technical efficiency of energy (sub-industry).

**Figure 19:**
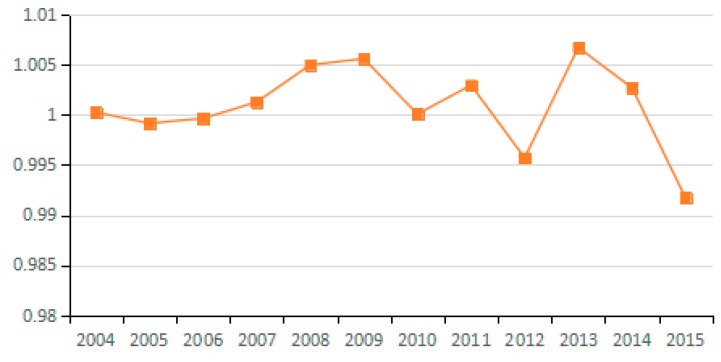
Changes in technical efficiency of energy (industry average change).

Based on the Shephard distance function, energy technology efficiency indicates the possibility of reducing energy consumption assuming other conditions remain unchanged. By analyzing the average value for energy technology efficiency of the 23 industries from 2003 to 2015, seven industries are identified as having reduced carbon emissions due to improved energy efficiency, and 10 industries have increased carbon emissions due to efficiency changes. As can be seen from the Figure 18, the factor of energy technology efficiency does not exert an obvious influence on carbon emissions because the values for the 23 industries fluctuate between 0.98 and 1.01. Moreover, by analyzing the average values for technological progress of the 23 industries from 2003 to 2015, the energy technology efficiency actually suppresses the reduction of carbon emissions over a long period (Figure 19).

Theoretically, the increase in energy efficiency is conducive to reducing carbon emissions. However, the results show that it does not play this role during the study period. To a certain extent, the absence of an obvious influence from this factor indicates that various industries do not attach enough importance to improving energy efficiency, and do not recognize the role of energy efficiency in energy conservation and emission reduction. This phenomenon is apparent in the secondary industry as well as in the tertiary industry. Hence, in view of the urgent need for energy conservation and emissions reduction, China should promote the improvement of energy efficiency as an important emissions reduction strategy.

(8) Energy Technology Progress Factor

**Figure 20:**
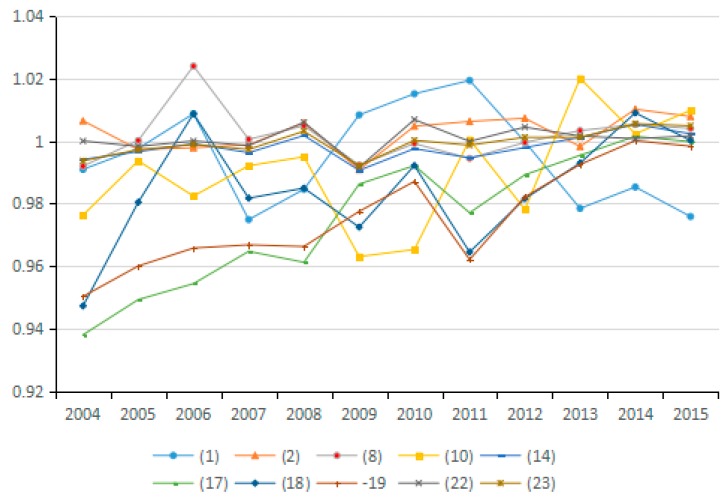
Changes in technical progress of energy (sub-industry).

**Figure 21:**
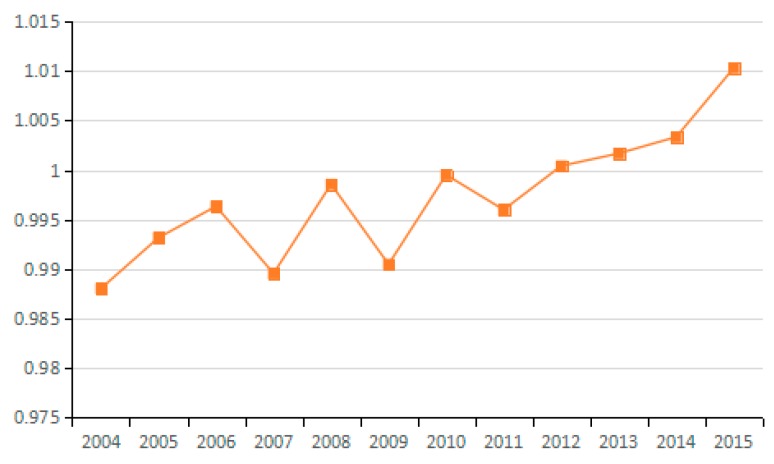
Changes in technical progress of energy (industry average change).

Decomposed by the Malmquist index, the energy technology factor measures the movement of the production frontier of all assessed industries from the *t* period to the *t* + 1 period with reference to energy consumption, and examines the progress of industries’ energy technology. Based on the results of factor decomposition, 10 representative industries were selected to show the directions of changes in the energy technology progress factor, meanwhile, the industrial average change value in technological progress was calculated (see Figure 20 and Figure 21). By analyzing the average value for the energy technology progress factor in the 23 industries from 2003 to 2015, 10 industries are identified that exhibit carbon emission reduction due to energy technology improvement, indicating that these industries have innovation capabilities or advanced energy technologies to a certain extent. In addition, 13 industries are identified that have increased carbon emissions due to technological improvements. Examining the 23 industries collectively shows that, during 2003–2011, energy technological progress played a positive role in reducing carbon emissions but the effect was relatively weak, and that carbon emissions increased after 2011. These results indicate that the innovative content of China’s energy technology is far from sufficient, and efforts should be made to improve the level of innovative technology and introduce advanced energy technologies.

(9) Technical Efficiency Factors of GDP Output

**Figure 22:**
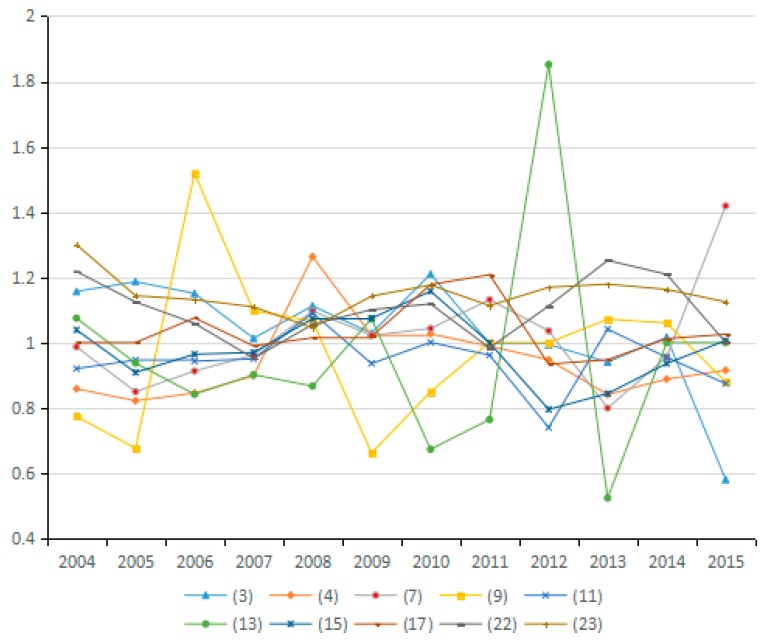
Changes in technical efficiency of GDP output (sub-industry).

**Figure 23:**
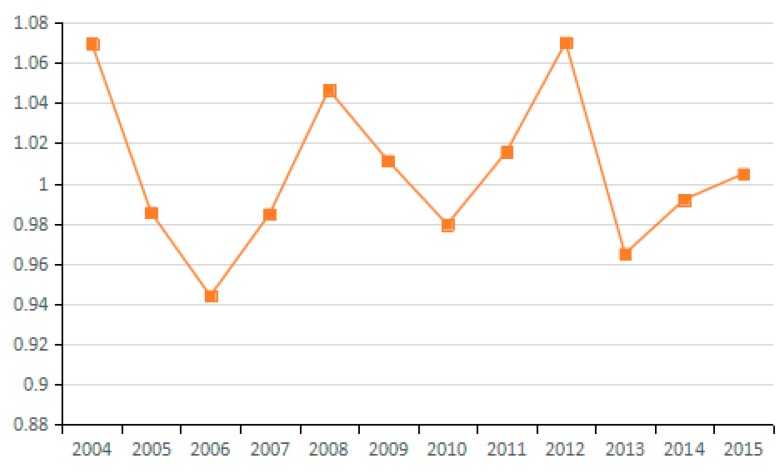
Changes in technical efficiency of GDP output (average change of industry).

Output technical efficiency was employed to measure the distance changes from an assessed industry to the optimal production boundary from the *t* period to the *t* + 1 period. Figure 22 shows the direction of change in the technical efficiency factors of GDP output in 10 representative industries, and Figure 23 presents the average value of changes in technical efficiency of all 23 industries. By analyzing the average values for technical efficiency of GDP output for the 23 industries from 2003 to 2015, eight industries are identified that reduce carbon emissions due to the increased technical efficiency of GDP output, among these, industry (4) and industry (13) show obvious effects. Likewise, nine industries are identified that have increased carbon emissions due to changes in technological efficiency; among these, industries (16), (22), and (23) present obvious effects. These results are consistent with the effects on carbon emissions of energy technology efficiency in different industries. Judging from the average value for the 23 industries, the effect of energy technology efficiency level on carbon emissions in 2003–2015 is not uniform because the output technical efficiency exhibits both negative and positive effects on increasing carbon emissions in different periods. The improvement of output technology efficiency should play a positive role in reducing carbon emissions. However, the study results suggest that the technical efficiency of almost half of the industries has not improved reasonably and there is still much room for improvement.

(10) Technological Progress of GDP Output Factor

**Figure 24:**
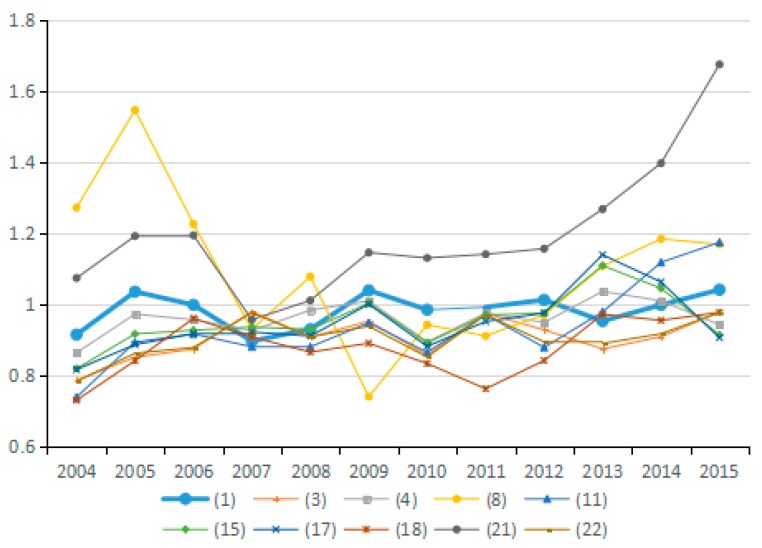
Changes in technical progress of GDP output (sub-industry).

**Figure 25:**
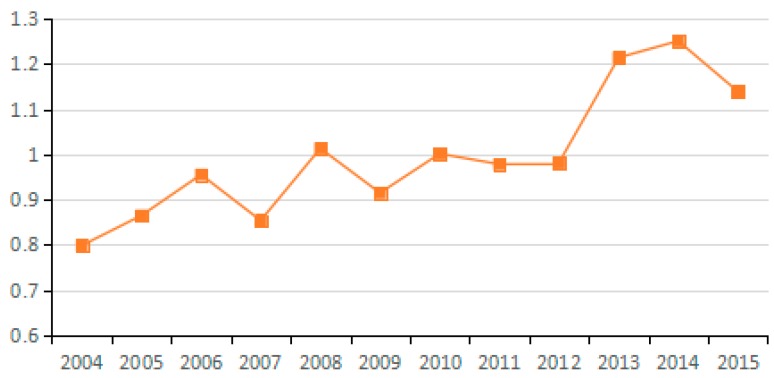
Changes in technical progress of GDP output (average change of industry).

Decomposed by the Malmquist index, the technological advancement factor of GDP output measures the movement of the production frontier of the assessed industries from the *t* period to the *t* + 1 period with reference to GDP output, and presents the progress of GDP output. By analyzing the average value of the technological advancement factor of GDP output for the 23 industries from 2003 to 2015 (see Figure 24 and Figure 25), 15 industries are identified with reduced carbon emissions due to GDP technological progress. This result indicates that the production frontier of the industries progresses with reference to GDP output, and the technological advancement factor of GDP output plays a positive role in carbon emission reduction. However, eight industries are identified that have increased carbon emissions due to technological advances. Examining the 23 sampled industries collectively shows that in the period 2003–2011 (excluding 2007 and 2009), technological progress in GDP output plays a positive role in reducing carbon emissions and that the effect is obvious, while in other periods, technological progress increases carbon emissions. For certain industries, technological progress is accompanied by greater carbon emissions, which indicates that China’s economic development still relies on a large input of resources. Therefore, it is imperative to promote the progress of production technology in view of the current extensive economic development.

## 5. Conclusions and Policy Implications

### 5.1. Conclusions

In this study, a factor decomposition model of carbon emissions for 23 groups of Chinese industries was constructed and 10 factors influencing the emissions were analyzed for the period 2003–2015. The factors are potential energy carbon intensity, energy mix, potential energy intensity, potential GDP, technological progress and technical efficiency of carbon emissions, energy technological advances and technologies efficiency, technological progress and technological efficiency of GDP output. The study results, reinforced by the fact that so many industries were examined over such a long period, support the following conclusions about the influence of various factors on industrial carbon emissions in China.

The potential GDP factor is important in causing the increase of carbon emissions and even determines the direction of carbon emission changes. The potential carbon intensity factor also causes increased carbon emissions due to the high carbon emissions from coal. The potential energy intensity factor exerts an obvious inhibitory effect on carbon emissions, as does the energy mix factor (albeit with a weaker effect). The effects of the technological advancement factor and the technical efficiency factor are inconsistent among industries. The technological advancement factor of GDP output inhibits carbon emissions to a certain extent.

The potential energy carbon intensity factor of most industries hinders the reduction of carbon emissions, showing that China’s adjustment of energy carbon intensity has not improved much during the 2003–2015 study period. Although the energy mix factor contributes to the reduction of carbon emissions, the effect is not obvious. The potential energy intensity factor contributes significantly to carbon emission reductions (the change values for this factor in most industries are <1). The potential GDP of most industries contributes to carbon emissions and is the most important positive contributor to carbon emissions. In most industries, the carbon emission technology efficiency factor increases carbon emissions. Therefore, there is still much room for China to improve its carbon emission efficiency level. 

Judging from the industry average, the technological progress factor can inhibit carbon emissions, however, energy technology efficiency does not play a corresponding role in carbon emission reduction. To a certain extent, the latter result indicates that various industries in China have not paid enough attention to improving energy efficiency and that energy efficiency has not been fully exerted to achieve energy conservation and emission reduction. Energy technological advancement plays a positive role in reducing carbon emissions yet with relatively weak effect. Technical efficiency of GDP output generally contributes to increased carbon emissions but sometimes inhibits the emissions. The technical efficiency of almost half of the industries does not ideally increase, leaving much room for improvement. At the early stage of industrial development, the technological progress of GDP output plays a positive role in reducing carbon emissions, while at the later stage, this factor increases carbon emissions.

### 5.2. Policy Implications

#### 5.2.1. Improving Economic Growth Quality and Balancing Economic Growth with Environmental Protection

Among the primary, secondary and tertiary industries in China, the secondary industry still occupies a large proportion. Hence, the proportion of domestic GDP contributed by secondary industry should be gradually reduced, and measures are to be implemented to vigorously encourage the rapid development of the tertiary industry, thereby rationalizing China’s industrial structure. The focus of these measures should be placed initially on industries with high consumption and high emissions to effectively integrate resources in these industries, phase out backward industries, and step up the development of industries with sound economic benefits and high environmental benefits. This strategy is also the main scope of China’s initiative in building a resource-conserving and environment-friendly and environmentally friendly society.

#### 5.2.2. Optimizing China’s Energy Mix and Vigorously Developing Clean Energy

Energy mix adjustment has a significant effect on reducing carbon emissions and is of great significance to China’s strategy of sustainable development. China is now gradually adjusting its energy development strategy and reducing its reliance on coal. However, as the effect of this adjustment is still not particularly obvious, it is necessary to increase the intensity of energy mix adjustment. There are two specific ways that this objective can be achieved. First, the proportions of the three major energy sources consumed in China should be adjusted by increasing the use of natural gas while reducing coal consumption. Second, “clean-energy” sources should be vigorously developed and the proportion of clean energy use should be increased. The carbon emissions from clean energies are negligible compared with those from fossil energies. Because China is rich in clean-energy sources such as water, solar energy and biomass energy, support and encouragement are to be given to the development of these clean sources of energy.

#### 5.2.3. Improving China’s Overall Innovation Capacity and Using Technology Changes to Reduce Carbon Emissions

Except for some industries, technical efficiency has caused an increase of carbon emissions instead of significantly reducing them. This fact indicates that the innovation capability of China needs to be improved from both input and output perspectives. Starting from all aspects of energy use, effective management of multiple aspects will promote energy efficiency. For most industries, technological progress plays a positive role in reducing carbon emissions, yet the effect is not dramatic. Therefore, improved energy technologies (such as clean utilization technology of coal) should be introduced and developed so as to achieve efficient energy utilization and reduce carbon emissions. Furthermore, to reduce China’s carbon emissions by promoting technological progress and improving technical efficiency, the government must first strengthen the country’s ability to innovate. This objective can be accomplished by intensifying cooperation between the government and enterprises, stimulating the technological innovation capabilities of enterprises, encouraging collaborative university research, and strengthening cooperation in advanced technology to promote technological progress. In addition, China should also actively introduce internationally used advanced technologies to reduce the indigenous research and development cost while improving domestic technologies.

#### 5.2.4. Improving Carbon Trading Market and Allocating Carbon Emission Quotas Efficiently and Fairly

In order to minimize pollution and maximize market benefits, the improvement of carbon market trading mechanism itself is a multi-objective decision-making problem. It is vital to solve this problem simply and effectively [55]. The allocation of carbon emission quotas should consider both efficiency and fairness [10]. The market property of carbon emission quotas can be regarded as a kind of scarce resource. To achieve the optimal allocation of scarce resources, not only financial indicators are to be considered, but also social, environmental and cultural factors should be taken into account [56]. Therefore, in the process of improving the carbon market, the government is to advance the transparency of trading information in the carbon trading market, promote the liquidity of the carbon trading market, and improve the level and ability of participants in the carbon trading market. In addition, it is of great importance to give full play to the functions of the government and pay attention to the influence of social, environmental and cultural factors in various regions.

## Figures and Tables

**Table 1:** Definition of variables.

Variable Symbol	Variable Name	Description
C	Carbon emissions	Calculate using standard coals with different energy sources and corresponding carbon emission factors
E	Energy consumption	Convert coal, oil, and natural gas into standard coal for totaling
Y	Economic scale	Actual industry added value in 1990 constant price
EM	Energy mix	Coal consumption/total energy consumption
EI	Energy intensity	Energy consumption/GDP
K	Capital stock	Cumulative capital of the industry
L	labor force	Average number of employees in the industry

**Table 2:** Sample industry.

Number	Industry	Number	Industry
(1)	Farming, Forestry, Animal Husbandry, Fishery Conservancy	(13)	Smelting and Pressing of Metals
(2)	Mining and Washing of Coal	(14)	Manufacture of Metal Products
(3)	Extraction of Petroleum and Natural Gas	(15)	Manufacture of Special Purpose Machinery and Manufacture of General Purpose Machinery
(4)	Mining and Processing of Metal Ores	(16)	Manufacture of Transportation Equipment
(5)	Mining and Processing of Non-metal Ores and Others	(17)	Manufacture of Electrical Machinery and Apparatus
(6)	Manufacture of Foods and Manufacture of tobacco	(18)	Manufacture of Computers, Communication and Other Electronic Equipment
(7)	Manufacture of Textile and Manufacture of products	(19)	Manufacture of Measuring Instruments and Machinery
(8)	Processing of Timber and Manufacture of Furniture	(20)	Production and Supply of Electric Power and Heat Power
(9)	Manufacture of Articles for Culture, Education, Arts and Crafts, Sport and Entertainment Activities	(21)	Construction
(10)	Processing of Petroleum, Coking and Processing of Nuclear Fuel	(22)	Transport, Storage and Post
(11)	Manufacture of Chemistry	(23)	Wholesale, Retail Trade and Hotel, Restaurants
(12)	Manufacture of Non-metallic Mineral Products		
